# Real-time molecular imaging of near-surface tissue using Raman spectroscopy

**DOI:** 10.1038/s41377-022-00773-0

**Published:** 2022-04-08

**Authors:** Wei Yang, Florian Knorr, Ines Latka, Matthias Vogt, Gunther O. Hofmann, Jürgen Popp, Iwan W. Schie

**Affiliations:** 1 Leibniz Institute of Photonic Technology Jena, Albert-Einstein-Straße 9, 07745 Jena, Germany; 2 Department of Trauma, Hand and Reconstructive Surgery, University Hospital Jena, Am Klinikum 1, 07747 Jena, Germany; 3 Institute of Physical Chemistry and Abbe Center of Photonics, Friedrich-Schiller University Jena, Helmholtzweg 4, 07743 Jena, Germany; 4 Department of Medical Engineering and Biotechnology, University of Applied Sciences - Jena, Carl-Zeiss-Promenade 2, 07745 Jena, Germany

**Keywords:** Raman spectroscopy, Imaging and sensing

## Abstract

The steady progress in medical diagnosis and treatment of diseases largely hinges on the steady development and improvement of modern imaging modalities. Raman spectroscopy has attracted increasing attention for clinical applications as it is label-free, non-invasive, and delivers molecular fingerprinting information of a sample. In combination with fiber optic probes, it also allows easy access to different body parts of a patient. However, image acquisition with fiber optic probes is currently not possible. Here, we introduce a fiber optic probe-based Raman imaging system for the real-time molecular virtual reality data visualization of chemical boundaries on a computer screen and the physical world. The approach is developed around a computer vision-based positional tracking system in conjunction with photometric stereo and augmented and mixed chemical reality, enabling molecular imaging and direct visualization of molecular boundaries of three-dimensional surfaces. The proposed approach achieves a spatial resolution of 0.5 mm in the transverse plane and a topology resolution of 0.6 mm, with a spectral sampling frequency of 10 Hz, and can be used to image large tissue areas in a few minutes, making it highly suitable for clinical tissue-boundary demarcation. A variety of applications on biological samples, i.e., distribution of pharmaceutical compounds, brain-tumor phantom, and various types of sarcoma have been characterized, showing that the system enables rapid and intuitive assessment of molecular boundaries.

## Abstract (web summary)

A fiber optic probe-based Raman imaging system with real-time molecular virtual reality data visualization of chemical boundaries for clinical tissue-boundary demarcation.

## Graphical abstract

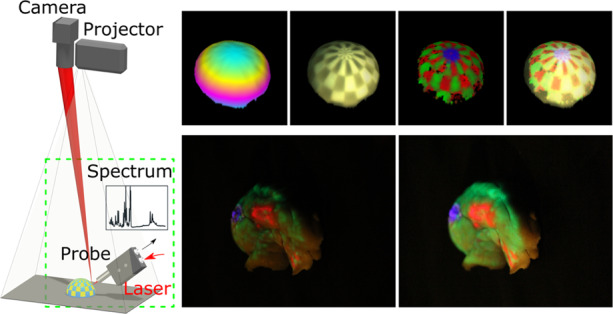

## Introduction

Methods, such as magnetic resonance imaging, computed tomography, positron-emission-tomography, and ultrasound are go-to tools that allow physicians to screen patients for cancer and localize suspicious lesions for surgical excision. These methods can also be functionalized, improving the precision for guided surgery, while allowing continuous and non-invasive monitoring of patients. In most cases, however, current imaging techniques provide information based on morphological or anatomic differences of the tissue, disregarding the underlying molecular composition. Recently, there has been a significant emphasis on Raman-based technologies for clinical in vivo applications. Raman spectroscopy is based on an inelastic scattering event between a photon and a molecule, providing the intrinsic molecular fingerprint of a sample. The advantage is that the information can be assessed label-free, without contact, and non-destructive. A number of previous studies have indicated that it is quite capable to detect and delineate cancer from healthy tissues^1–8^. However, the current implementation of fiber optic probe-based Raman spectroscopy stays well behind the technological possibilities the method can offer. To explore the potential of the method, multiple challenges have to be addressed, including imaging acquisition, sample topology, and real-time data analysis. The solution will also result in new opportunities, such as data visualization and comprehension, which will have to be explored.

For in vivo applications fiber optic probe-based Raman systems are used, because these probes are small, flexible, and allow direct access to the patient site^9–12^, see Supplementary Table S1. Currently, Raman measurements with fiber optic probes are performed from individual locations of the sample, representing the molecular information from the respective focal volumes. Because the measurement position of a probe is not co-registered with the measurement information it is currently not possible to create an image using a handheld fiber optic Raman probe. To enable image formation, approaches using multiple optical fibers have been suggested^13^. This allows to sample multiple points at once to create a low-resolution image from a limited area but requires the application of a very large power-density on the tissues, making them less favorable for clinical translation. For large-scale imaging, a physical tracking of the probe movement to relate the positional information with the measurement location can be achieved using passively coordinated mechanical arms^14,15^ and robotic arms^16^. This, however, is not only bulky but also increases unnecessarily the complexity of a system. Rather than using physical tracking, computer vision-based positional tracking approaches can provide a suitable alternative. Those operate with conventional imaging detectors to track the position of an aiming laser, avoiding complex physical instrumentations, and enabling more flexible handling, while providing positional registration with the sample^17–19^.

Besides no real imaging capability for fiber optic Raman probes, current Raman systems only acquire the data but do not provide instantaneously the diagnostic information, and the collected data are analyzed post-acquisition, reducing the benefit of the method. While some implementations provide online analysis^20,21^, they do so for single-point measurements and cannot be used for applications, such as tumor margins detection. However, if the acquisition of Raman images with a handheld probe was possible, the combination with real-time data processing could enable the visualization of the molecular distribution in clinical applications. This could also be favorably combined with augmented reality (AR) and mixed reality (MR) to enhance the perception of molecular information. These virtual reality (VR) approaches offer an interesting potential to improve the precision of real-time diagnosis or surgery^22^ and have readily been combined with various imaging modalities, such as computed tomography^23^, optical coherence tomography^24^, magnetic resonance tomography^25,26^, fluorescence lifetime imaging^17,19^, and others^18,19,27,28^.

To overcome the aforementioned challenges, we propose and experimentally demonstrate a fiber-based Raman imaging approach with real-time data analysis and combination with augmented and mixed reality on three-dimensional (3D) sample surfaces as a potential tool for real-time opto-molecular visualization of tissue boundaries for disease diagnostics and surgical resection. The reported imaging platform combines Raman spectra measurements, simultaneous computer vision-based positional tracking with real-time data processing, and real-time formation of molecular virtual reality (MVR) images. The MVR images can be perceived on the computer screen or, by additionally using a laser projector system, directly mapped on the tissue, creating MR information that can be perceived by the naked eye in real-time. Because most samples have a topological surface profile, we have additionally implemented a photometric stereo measuring system, which allows mapping the molecular information on a 3D sample surface. The presented work outlines the potential for future clinical translation of real-time Raman-based molecular imaging, by allowing easy access to patients and by providing biochemical distributions from the region of interest for disease tissue differentiation during surgical resection.

## Results

### Imaging of a 3D structured bio-sample surface and ex-vivo tumor tissue

**Fig. 1:**
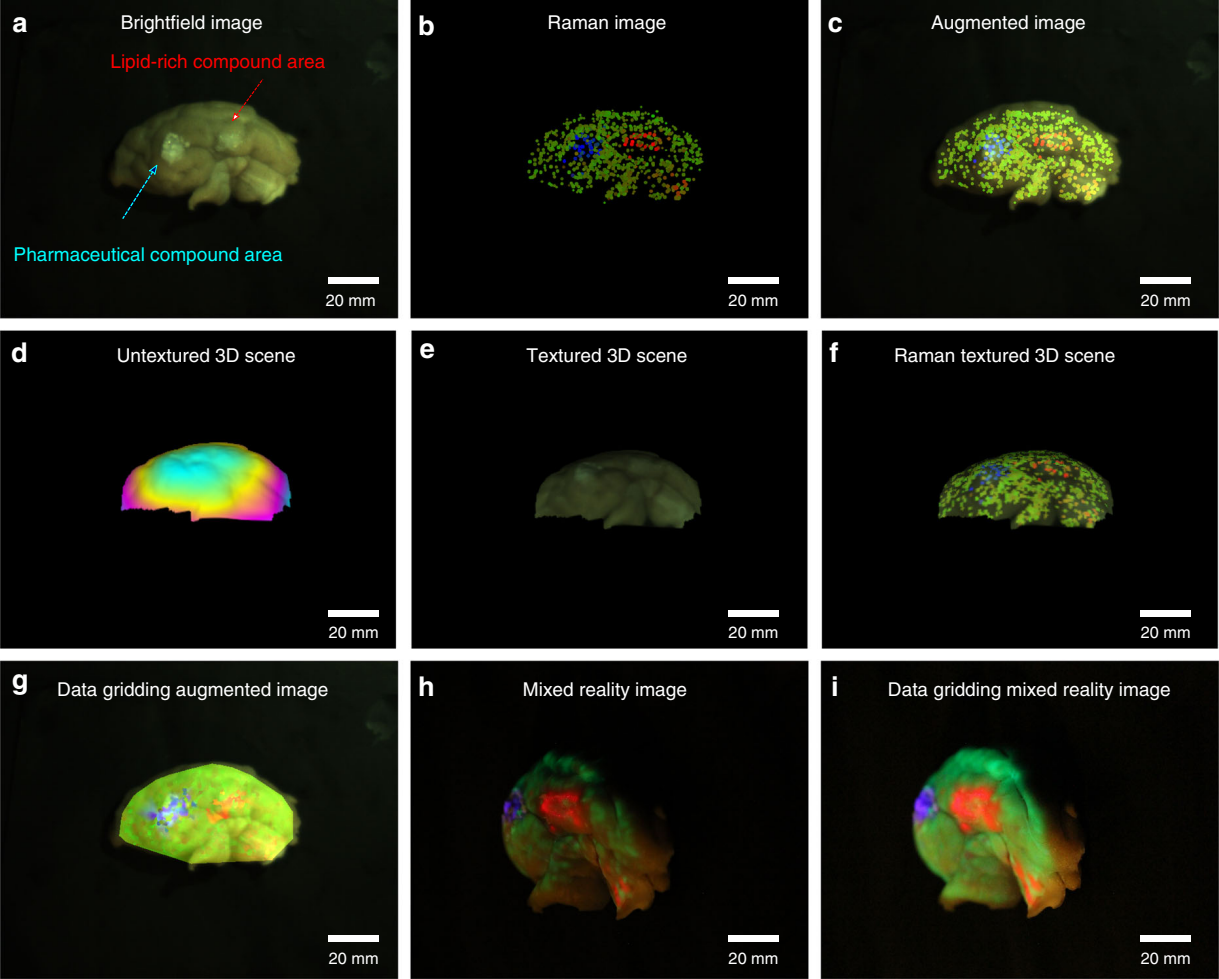
Representative results of the proposed approach on a porcine brain sample. **a** Brightfield image of the sample, where the arrows indicate areas with a lipid-rich and pharmaceutical compound. In the brightfield image it is not only challenging to localize the regions but does not provide any possibility to differentiate between regions. **b** Reconstructed Raman image provides the molecular distribution on the sample. **c** Augmented reality image directly combines the molecular information with the brightfield scene, readily providing a differentiation between the regions. **d** The reconstructed untextured height 3D scene image of the sample employing photometric stereo and **e** as textured 3D image with the original brightfield image. **f** The height 3D scene image textured with the reconstructed Raman image, which now not only enables the two dimensional, but three dimensional visualization of the augmented molecular information. **g** Raman image after data gridding of the molecular data, forming a fully filled 3D augmented molecular image. **h** Improved and direct visualization of the mixed Raman reality image is back-projected onto the sample, so that the molecular boundaries are visible to the user. **i** Presents the results as in **h**, but with gridded data, which allows to better visualize molecular boundaries. The color information in all figures represent the different chemical components, i.e., red for the lipid-rich compound, green for gray matter on the cerebrum, and blue for the pharmaceutical compound

To evaluate potential biomedical applications porcine cerebrum and sarcoma tissue were used to differentiate molecularly distinct regions with the proposed approach. A porcine cerebrum with dimensions of 7 cm × 4 cm × 2.6 cm was used. To simulate heterogeneous regions two areas of the cerebrum were coated with a lipid-rich and a pharmaceutical compound (N-Acetyl-4-aminophenol). The brightfield image of the prepared sample is presented in Fig. 1a and reference spectra from both compounds and the gray matter are presented in Supplementary Fig. S1. The laser power and acquisition time was set to ~100 mW and 0.1 s, respectively. The brain surface was scanned by hand with the outlined approach and in 260 s a total of 856 points were sampled, processed, and reconstructed, Fig. 1b, c. To visualize the topological distribution of molecular compounds photometric stereo was used and the non-textured height image and the textured image with the original color image are presented in Fig. 1d, e, and the molecular information mapped onto the reconstructed 3D model, Fig. 1f and gridded molecular data, Fig. 1g. The combination of the molecular information with the photometric stereo offers an intuitive and realistic view of the distribution of the chemical information on the surface in a 3D scene compared to the conventional 2D image. The 3D representation of the structured sample was recorded and displayed in individual panels of non-textured height image, original color textured image, and Raman textured image, in Supplementary Video 1a. The MR-representation is presented in Fig. 1h, i, where the users can intuitively observe the distribution of the components directly on the sample. The video recordings of the acquisition-procedure, including augmented and mixed reality visualizations can be found as Supplementary Video 1b. As shown in Supplementary Fig. S1c, the detection of very low concentrations can be challenging. While the current implementation uses compounds at a high concentration, future modifications of the optical parameters of the acquisition system will additionally improve the performance, which will enable measurements at very low concentrations.

**Fig. 2:**
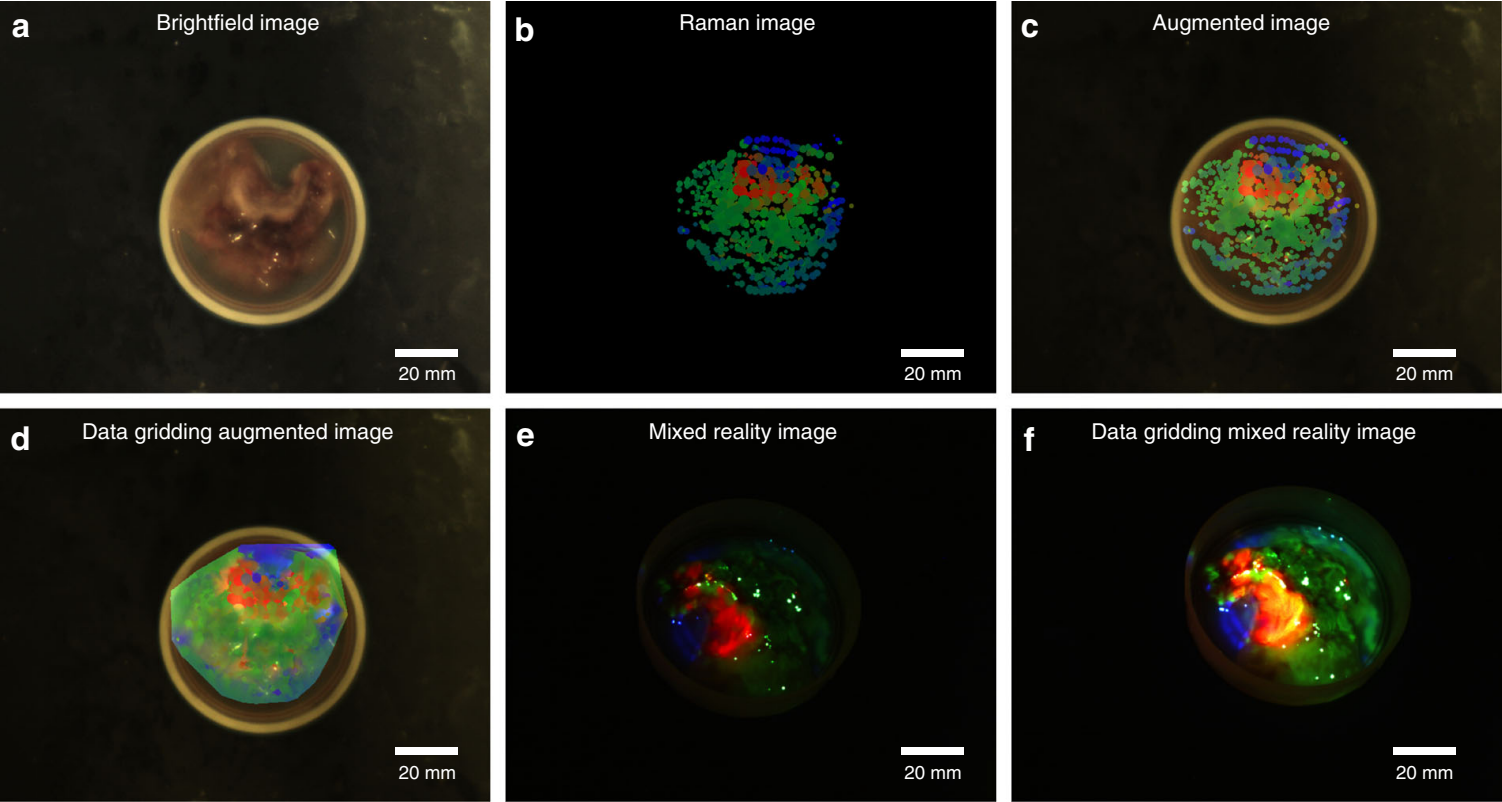
Representative results of the proposed approach on the ex-vivo clinical sample. **a** Brightfield image of the sample does not allow to differentiate between different molecular regions. **b** Raman imaging with the presented approach readily enables the visualization of distinct molecular locations. **c** The augmentation of the molecular information with the brightfield image provides distinct differentiation of the information of the sample. **d** Data gridding of molecular information and augmented overlay with brightfield image. **e** Mixed Raman reality image enabled through projection the direct visualization of the molecular information in the sample plane; and, in combination with data gridding (**f**), provides a rich image with molecular distinct boundaries. The color information represents the different chemical components, i.e., red for collagen, green for epithelial tissue, and blue for the plastic sample holder

To demonstrate the performance of the reported approach for the tumor characterization, a measurement on a freshly excised tumor sample with a size of 5.2 cm × 4.1 cm was performed. The laser power and acquisition time was set to ~100 mW and 0.1 s, respectively. By scanning the sample surface with the handheld movement of the probe a total of 994 points were acquired in 184 s, and the corresponding brightfield image, Raman image, the augmented image, and the gridded are presented in Fig. 2a–d, respectively, showing distinct molecular boundaries within the pathological tissue. The reference spectra can be found in Supplementary Fig. S2. Using the mixed reality visualization method, the molecular information was projected on the sample surface and recorded with an external DSLR camera (digital single-lens reflex camera, EOS 1200D, Canon, Japan), Fig. 2f, g. Due to an increase of sample transparency, the mixed Raman reality was not real-time updated, but only the final molecular image projected on the sample. The video of the whole procedure of the Raman reconstruction with the augmented method can be found in Supplementary Video 2. Additionally, measurements on ex vivo cancer lipoma tissue and the results can be found in Supplementary information of Fig. S3 and Video S1. In all examples the presented implementation is able to differentiate between various biomedical components during the data acquisition process, resulting in a real-time assessment of the macromolecular distribution of the sample, opening new potentials for image-guided in-vivo disease diagnostics and surgical resection.

### Data-flow and diagram

**Fig. 3:**
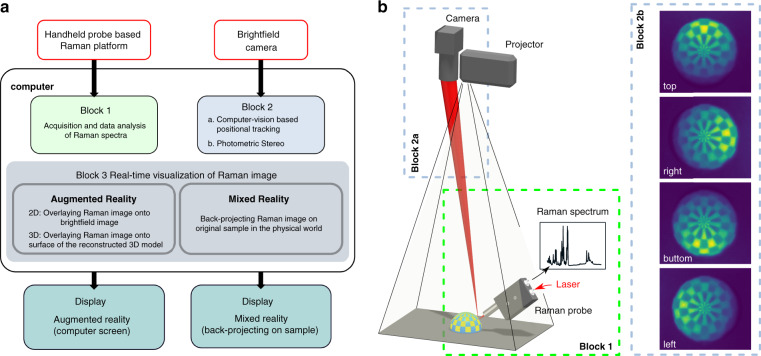
Data-flow (a) and diagram (b) of the developed approach. Raman spectra are acquired with a handheld Raman probe (Block 1) and positional information where the spectra were acquired is determined in parallel through a brightfield camera (Block 2a). The Raman spectra are processed in real-time and combined with the positional information to reconstruct the Raman image, forming the molecular virtual reality visualization of the molecular distribution. Furthermore, a computer vision-based 3D surface reconstruction method (Block 2b), i.e., photometric stereo, is used to construct a 3D height map of the sample surface, allowing to overlay the reconstructed Raman image on the 3D map for a 3D visualization of the molecular composition

The data flow and diagram of the implementation are presented in Fig. 3, where the first part focuses on parallel running spectral acquisition and simultaneous positional tracking, while the second part on the topographic reconstruction, real-time Raman-data analysis, and the molecular visualization using AR and MR. In AR the molecular information is mapped on the brightfield image or a 3D surface model formed by the photometric stereo method on the computer screen. In MR the molecular information is projected on the sample. In our implementation we register the projected image with the sample in the following way:

**Block 1**: As described in ref. ^18^, a handheld fiber-optic Raman probe is used to acquire Raman spectra. First, a database of reference spectra from the relevant components of the sample is built. For bio-sample, this database contains Raman spectra of basic molecular components, such as lipid, bone, protein, collagen, and so on. Additional reference-spectra can be added to the database by sparse Raman sampling on the target sample. Each newly acquired spectrum is baseline corrected, using an ALS (alternating least squares) -based background estimation and normalized to unity^29^. The corrected and normalized spectra are then fitted using the database-spectra by non-negatively constrained least squares fitting^30^:

1 $${{{\mathbf{s}}}} = {{{\mathbf{Pc}}}} + \varepsilon$$

where **s** is an acquired spectrum, **P** is the database spectra, **c** a coefficient vector with the length of the vector equals the number of spectra in the components database, representing the relative concentration of the components and *ε* is the error of the measurement. By solving the equation under the non-negativity constrain for the coefficient values, the vector result **c** is used to construct molecular concentration maps for the database spectra.

**Block 2: a**. During the Raman measurement, the brightfield camera video-stream is used to identify the current measurement location through the analysis of the excitation laser point generated by Rayleigh scattered light. The image processing is based on color-segmentation, thresholding, and ellipse fitting during the measurement. It should be pointed out that the threshold in this step cannot only ensure that the bright beam generated by the Rayleigh scattered light is retained, but also helps to avoid a certain degree of defocused measurement. Because in case of the strong defocusing, the intensity of the laser beam will be too low for the intensity threshold, resulting in failure of subsequent ellipse fitting and the measurement is discarded. If the fitting is successful, the parameters of the fitted ellipse, i.e., center, and minor radius are used for Raman image reconstruction. **b**. Photometric stereo, based on four light sources, is integrated into the reported approach to reconstruct the 3D surface of a sample. This method is applied prior to the Raman measurements to build a 3D surface model of the sample, which is then used to map the extracted molecular distribution on the 3D model of the sample.

**Block 3:** The next steps concern the visualization of the Raman information, both as augmented and as mixed reality. For each new frame, a circle with an auto-scalable diameter is computed in the 8-bit intensity image of each known components plane. The center point of the circle is equal to the center point determined by laser tracking (Block 2), whereas the concentration coefficient is determined by the non-negative estimation (Block 1). The scalable diameter (*d)* of the circle is determined by the minor radius (*r*) of the fitted ellipse and the Euclidian distance (*D*) between the center of the current fitting ellipse and the one from the previous frame as the following rule:

2 $$d = \left\{ {\begin{array}{*{20}{l}} {0.5r,\;{\mathrm{if}}\;D \,<\, r} \\ {r,\quad\;\,\,{\mathrm{if}}\;r \le D \,<\, 2r} \\ {2r,\quad\;{\mathrm{else}}} \end{array}} \right.$$

For each element of the database, an image plane is updated according to:

3 $$I_{{\mathrm{x,y}}}^{\mathrm{k}} = \frac{{{\sum} {c_{{\mathrm{x,y}}}^{\mathrm{k}}} }}{{N_{{\mathrm{x,y}}}}}/c_{{\mathrm{max}}} \times 255$$

where for the image plane of the *k*th component, at the pixel location (*x, y*), the intensity $I_{{\mathrm{x,y}}}^{\mathrm{k}}$ is determined by the mean value of the relevant non-negative estimation result ${\textstyle{{{\sum} {c_{{\mathrm{x,y}}}^{\mathrm{k}}} } \over {N_{{\mathrm{x,y}}}}}}$. Here, *N*_x,y_ is the number of measurements at the pixel (*x, y*), which is then normalized to the maximum value of all the fitting result *c*_max_, and rescaled into an 8-bit (0–255) scale. Additionally, a data gridding function, using the triangulation-based nearest-neighbor interpolation method can be used^31^. This method is applied to individual 8-bit intensity images of component planes and spatially interpolates randomly distributed data points over a uniform grid. Here, the grid size is set to the image resolution and can be adjusted as needed. At last, an RGB-image is displayed by merging the three pseudo-color planes. Because the Raman image is reconstructed on the same dimensional space as the brightfield image, it is easy and convenient to overlay the molecular distribution on the brightfield image to display the augmented chemical reality on the computer screen in real-time. This overlay can be performed with both 2D and 3D models, whichever is most appropriate for the current sample.

To overcome the need to change viewing directions during surgery is the use of mixed reality. We additionally implemented spatial mixed reality by projecting the molecular information in real-time during the acquisition onto the tissue, using a small-size laser projector (BML100PI, Bosch, Germany). In order to avoid disturbances of the laser tracking, the intensity of the projected image is reduced by adjusting the transparency.

### Characterizing the instrumentation

#### 3D reconstruction

**Fig. 4:**
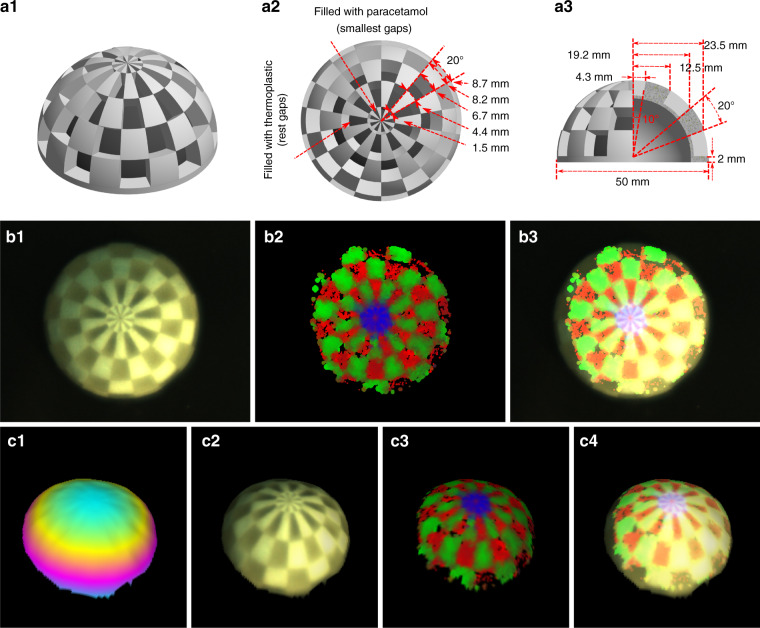
Combination with the photometric stereo. **a**_**1**_ Diagram of the PMMA hemisphere phantom with 20°-slices from the center on the horizontal plane and vertical plane. The spaced area on the top surface is hollowed and the small gaps refilled with paracetamol, while the remaining gaps are filled with moldable thermoplastic (polycaprolactone). **a**_**2**_ Top-view of the phantom, which shows the arc lengths of a 20° slice on each horizontal level. **a**_**3**_ Three-quarter side-view of the phantom, which shows the radiuses of each horizontal level. The diameter of the hemisphere is 50 mm and the thickness of the basement is 2 mm. **b**_**1–3**_ Top-view on the sample. **b**_**1**_ Brightfield image of the hemisphere phantom, where no molecular boundaries can be discerned. **b**_**2**_ Reconstructed Raman image by the developed imaging approach with the handheld operation with clear molecular boundaries, and **b**_**3**_ overlayed with the brightfield image as augmented molecular information. **c**_**1**_ A representative image of the height map of the phantom through photometric stereo. **c**_**2**_ The brightfield image textured height map, viewed from the same angle, and **c**_**3**,_ with relevant molecular-textured 3D image. **c**_**4**_ The three modes combined and represented as 3D augmented molecular image. The color information represents the different chemical components, i.e., red for PMMA, green for thermoplastic (polycaprolactone), and blue for paracetamol

Because in most relevant clinical applications the sample has a topographic profile, we have added photometric stereo to the implementation. To characterize the combination of the proposed Raman imaging approach with the photometric stereo method, a hemisphere phantom was designed and fabricated by 3D printing of Poly (methyl methacrylate) (PMMA), which has slices of every 20° from the center on the horizontal plane and vertical plane, respectively, Fig. 4a_1_. Relevant parameters can be found in the top-view, Fig. 4a_2_, and three-quarter view, Fig. 4a_3_. The basic implementation of the proposed Raman imaging method only allows the reconstruction of information from 2D surfaces, meaning that the information of the present phantom is a projection of the topography to the camera plane, resulting in a compression of the individual points at lower heights, Fig. 4a_2_. The laser power and acquisition time was set to ~100 mW and 0.1 s, respectively. The results for the molecular imaging without the topographic assessment are presented for the brightfield image, Raman image, and augmented Raman image in Fig. 4b_1–3__,_ resulting in deviations for tiles lengths located closer to the equator. To reduce these deviations photometric stereo was implemented on the same system, allowing to determine the topological height information and enabling to establish the molecular and height information and a 3D visualization of the molecular signatures. The 3D-surface model of the hemisphere phantom, Supplemental Fig. S4, shows a root-mean-square error (RMSE) and normalized root mean square error (NRMSE) between reconstructed height map and real height map of ~11.9 pixels for ~2.8 mm, corresponding to 0.24 mm/pixel and 10.6%, respectively. The reconstructed 3D image of the hemisphere phantom is shown in Fig. 4c_1_ and Fig. 4c_2_ with the untextured height scene and textured scene with the brightfield image. The untextured height scene is formed through a mapping of the height information into a CMY (cyan, magenta, and yellow) color scale, while the textured scene was formed by mapping the brightfield image on the height 3D model according to the corresponding pixel coordinates. Because the brightfield image is correlated with the acquired Raman information it is straightforward to map the molecular information on the 3D-surface, forming AR images, Fig. 4c_3_, c_4_. A video showing the 3D view representation of these images, Fig. 4c_1–4_, is found as Supplementary Video 3.

**Fig. 5:**
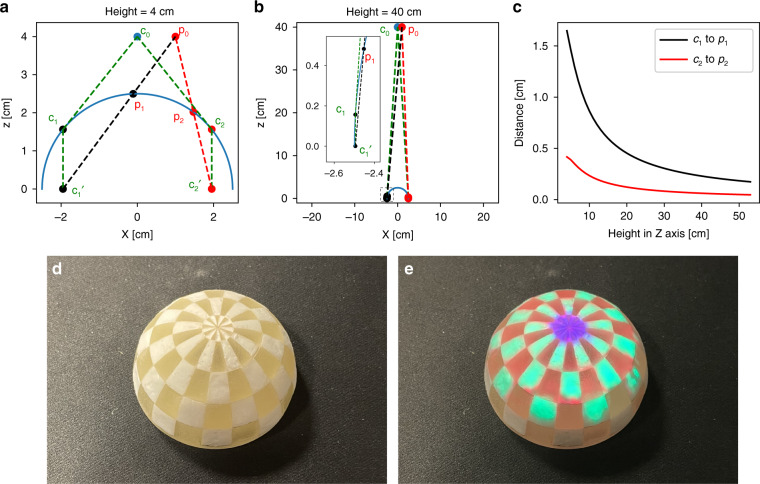
Characterization of the performance of mixed Raman reality for the hemisphere phantom. **a** Schematic diagram for the implementing of mixed Raman reality with the hemisphere phantom. Here, taking the section of *Y* = 0 into account, *c*_0_ and *p*_0_ represent the position of camera and projector, respectively. The horizontal distance between them is fixed to 1 cm. *c*_1_ and *c*_2_ are the tangent points from *c*_0_ to the phantom. *c*_1_′ and *c*_2_′ is the base perpendicular from *c*_1_ and *c*_2_ at *Z* = 0 axis. *p*_1_ and *p*_2_ are the intersections of the hemisphere with the segments of *p*_0_ to *c*_1_′ and *p*_0_ to *c*_2_′. **b** Same consideration for the arrangement, but at a height of 40 cm, where the subimage is the zoom-in of the small black-dashed box. **c** Height plot in *Z* axis vs. the distance between *c*_1_ to *p*_1_ (black curve) and *c*_2_ to *p*_2_ (red curve). **d** Photograph of the hemisphere phantom; and **e** photograph of the mixed molecular reality image by projecting the molecular image on the phantom. The color information represents the different chemical components, i.e., red for PMMA, green for thermoplastic (polycaprolactone), and blue for paracetamol

Because the homographic relationship can only be applied for two planar surfaces, the representation of the mixed chemical reality of the projection of the Raman image on the 3D surface would result in distortions between the projection and the original surface, Fig. 5a. For the phantom sample, taking the section of *Y* = 0 into account, *c*_0_ and *p*_0_ represent the position of the camera and projector, respectively. Here, the horizontal distance between camera and projector is fixed to 1 cm, where *c*_1_ and *c*_2_ are the tangent points from *c*_0_ to the phantom, and the cone spanned by these lines represents the FOV of the camera on the phantom. *c*_1_′ and *c*_2_′ are the foot of the perpendicular from *c*_1_ and *c*_2_ to *X*-axis. If the camera-projector calibration is based on the surface at *Z* = 0, *c*_1_′ and *c*_2_′ would be the projected points for *c*_1_ and *c*_2_. Thus, the projection points would be distorted, i.e., from *c*_1_ and *c*_2_ to *p*_1_ and *p*_2_, which are the intersections of the segments of *p*_0_ to *c*_1_′ and *p*_0_ to *c*_2_′ with the phantom, respectively. Exemplary, Fig. 5a, b show the situation for camera/projector distances to the sample of 4 cm and of 40 cm, respectively. The subimage is the zoom of the small black-dashed box to show the details for Fig. 5b. Based on this simulation, a plot of height in the *Z*-axis vs. the distance between *c*_1_ to *p*_1_ (black curve) and *c*_2_ to *p*_2_ (red curve) is shown in Fig. 5c. The distortions decrease with increasing height, if the thickness of the sample is fixed. For the current implementation, the height of the camera and projector to the sample was set to 40 cm, resulting in distortions between approx. 0.23 and 0.06 cm. Figure 5d, e show the photographs of the hemisphere phantom and mixed Raman reality by projecting the Raman image on the phantom. Since the PMMA is relatively transparent, the red color overlay is not obvious. The result also shows that the experimental distortions agree with the theoretical simulation, which indicates the distortions could be minimalized by increasing the ratio between the height of the projector (camera) and the thickness of the sample.

#### Spatial resolution

**Fig. 6:**
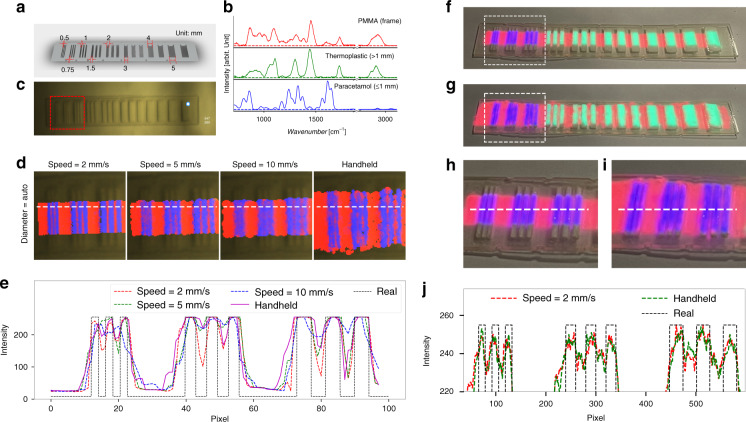
Evaluation of the spatial resolution of the developed approach. **a** Diagram of the spatial resolution target with stripes of various widths. The gaps between the stripes are filled with moldable thermoplastic (polycaprolactone) and paracetamol powder for gaps >1 mm and ≤1 mm, respectively. **b** Raman spectra of the relevant three components are color-coded, i.e., red for PMMA, green for thermoplastic, and blue for paracetamol. **c** Brightfield image of the spatial resolution target and an indicator of the ellipse fitted laser spot with positional information. The red dashed box indicates the ROI for imaging cropping. **d** The cropped augmented Raman images in the ROI of the auto-scale reconstructed diameter of the circles by moving probe at various speeds or handheld operation. The white dashed line on each image indicates the line for profile analysis. **e** The plots of the blue path (paracetamol plane) of the lines profile from the individual augmented Raman images. The black dashed line indicates the real region of the gaps filled with paracetamol. **f** Photograph of the mixed Raman reality result by projecting the molecular image reconstructed for the speed moving speed of 2 mm/s and the auto-scale diameter condition; and **g** for handheld operation. The white dashed boxes in the images indicate the ROI shown in detail in **h** and **i** and the corresponding intensity profiles of the dashed white line are presented in **j**. The real curve (black) indicates the gaps where the paracetamol is filled in

The auto-scale diameter rule of Eq. (2) was characterized to demonstrate the relationship between the spatial resolution of the Raman imaging system with the speed of the probe movement and the diameter of the reconstructed circle. Theoretically, the spatial resolution of the system is defined by the spatial resolution of the brightfield camera and the size of the laser spot. Here, the resolution of the brightfield camera is set to 640 × 512 pixels, and the field of view is approx. 156 mm × 125 mm, resulting in approx. 0.24 mm per pixel. The focal spot diameter of the laser is close to 105 µm, as in the fiber optic probe the excitation fiber with a core diameter of 105 µm is imaged by the optical system with a 1:1 magnification. As such, only the resolution of the brightfield camera and motion speed of the probe limits the resolution of the system. The projector used for the MR has a format of 854 × 480 pixels, which is similar to the one of the brightfield camera. However, for each frame, the reconstruction of the Raman image is based on the scalable circle, which is related to the minor radius of the fitted ellipse as mentioned above. The ellipse fitting method requires at least five points to perform the fitting^32^, i.e., at least a diameter of two pixels for the fit. The reconstruction requires minimal ±1 pixel surrounding the center position to draw the constructed circle, which sets the diameter of the reconstructed circle to at least two pixels. Thus, the theoretic spatial resolution of the whole system is approx. 0.5 mm in x-direction and y-direction. To assess these considerations a molecular sensitive spatial resolution target with stripes of different widths was designed and 3D printed, Fig. 6a. The widths of stripes and grooves vary from 0.5 mm to 5 mm. The molecular sensitive spatial resolution target is made of PMMA and the grooves between stripes are filled with moldable thermoplastic, i.e., polycaprolactone for gaps >1 mm and powdery paracetamol for gaps ≤1 mm. The Raman spectra of these three components are plotted as Fig. 6b, which were used as the database for the data evaluation. Pseudo colors were assigned to the individual components, i.e., red for PMMA, green for thermoplastic, and blue for paracetamol. Figure 6c shows the reference brightfield image of the resolution target and an indicator of the ellipse-fitted laser spot. To demonstrate the influence of the moving speed of the probe, a translation stage (MLS203–1, Thorlabs, Austin, Texas) was used. The probe was attached at a slight angle to ensure that the laser spot is not obscured. The speed of the stage was set to 2 mm/s, 5 mm/s, and 10 mm/s, respectively. The distance between probe and sample was to the focal distance of 8 mm. Also, a handheld operation for the probe movement was performed for comparison. For these measurements, the laser power and the acquisition time were set to 100 mW and 0.1 s, respectively. The images of visualization by AR method for 2, 5, and 10 mm/s and consequently different reconstructed circle diameters are shown as Supplemental Fig. S5. The reconstructed Raman image fits the known distribution and spacing of molecular compounds. To better visualize these details a region of interest (ROI), indicated by the red dashed box in Fig. 6c, was selected. Images were cropped and are presented in Supplemental Fig. S6, which shows that a small diameter of the reconstructed circle at a high speed of movement may lead to a sparse image, while the big diameter may result in a too smooth image with a decreased spatial resolution. The results show that the auto-scale rule, Eq. (2), can improve visual perception by dynamically adjusting the reconstructed diameter according to the moving speed. To demonstrate the details of the auto-scale results, the relevant cropped images with various moving speeds and the handheld operation by auto-scaling reconstructed diameters are presented in Fig. 6d. To visualize the contrast between the molecular components the intensity profile along the horizontal line (white dashed line) for the paracetamol (blue) plane on the individual images was plotted for different motion speeds, Fig. 6e. These images and the plots demonstrate that the auto-scale reconstruction strategy can strike a balance between the speed of the probe movement, and the quality of the reconstructed Raman image. It allows coarse-mapping at a higher speed to cover a larger image area, but reduced resolution and fine-mapping at low speed to acquire high resolution, especially benefiting the handheld operation. The plot also shows that the experimental spatial resolution agrees with the theoretical analysis. This means that a spatial resolution of approx. 0.5 mm can be achieved. The spatial resolution was also investigated for MR, by back projecting the reconstructed Raman image on the resolution target. However, beforehand the projector-camera was calibrated to establish the homographic relationship between the projector and the camera, i.e., the projective transformations between the camera plane and the projector plane, using a checkerboard. For details see supplementary information Figs. S7 and S8. With this calibration, the spatial mismatch error is around 0.3% or 1.5 pixels. Here, representative results of the movement speed of probe at 2 mm/s and handheld operation, both with auto-scale reconstructed circles were projected on the original resolution target, respectively, and photographed by the external DSLR camera, see Fig. 6f, g. The white dashed boxes in the photographs indicate the ROI which forms Fig. 6h, i, respectively. Figure 6j shows the horizontal line profiles of the paracetamol (blue) plane of the white-dashed lines on Fig. 6h, i with red-dashed curve and green-dashed curve, respectively. The real (black-dashed) curve represents the gap area where the paracetamol is filled in. The plot demonstrates that the mixed reality visualization has the same positional accuracy and spatial resolution as augmented reality. The achievable spatial resolution is comparable with conventional medical imaging methods, e.g., CT and MRI^33,34^, and should be suitable for the scenario as an image-guidance instrument for clinical applications. The spatial resolution could be improved with a higher resolution brightfield camera and smaller laser spot. In return, this would increase the measurement time for the same fill factor. The current parameters are a compromise between the time consumption for covering a sufficiently large area and suitable image quality.

## Discussion

Our proposed and experimentally demonstrated fiber-optic probe-based imaging system enables the non-destructive acquisition of molecular images from a large tissue sample label-free. To overcome the current disadvantage of conventional Raman systems, we have directly implemented the data-processing engine into the acquisition flow, enabling the evaluation of complex biochemical macromolecules Raman signatures in real-time and visualization molecular virtual reality, i.e., augmented reality and mixed reality. Additionally, for the application to 3D surfaces, an assessment of the topography through photometric stereo was implemented, allowing the mapping of the reconstructed molecular information on the 3D model of the sample. In the current configuration, the system achieves a spatial resolution of 0.5 mm, which is already below the typical excision precision of a surgical procedure, Fig. 3. Depending on the particular application acquisition and mapping frequency of 10 Hz can be achieved for excitation powers, which are below the minimally permittable exposure (MPE) value for radiation of for a 785 nm excitation, i.e., approx. 159 mW for 1 s, making this quite feasible for clinical translation. The photometric stereo can be determined with a normalized root-mean-square error (NRMSE) of only 3.3% in depth, corresponding to less than 1 mm in depth, also showing compatibility for clinical translation. The characterization of augmented reality and mixed reality on the resolution target and the hemisphere phantom for the situation of flat sample and 3D structure sample, show that depth distortions can be as low as 0.6 mm for a projector to sample distance of 40 cm. The application to the biological tissue phantom has shown the distinct differentiation of the molecular margins between the distribution of a lipid-rich and pharmaceutical compound on a 3D-structured porcine cerebrum. Through the implementation of photometric stereo, it is possible to precisely determine the distribution of the molecular composition on the tissue, enabling evaluation of the position of molecular margins and the analysis of two ex-vivo biopsy samples, breast cancer, and lipoma. Future improvements of the system will aim at a more precise probe movement and control of the sample to probe distance, i.e., combination with a rapid and accurate autofocus unit^35^. Additionally, we envision the combination or the translation to other optical modalities, such as optical coherence tomography (OCT), hyperspectral imaging, second harmonic generation (SHG), and others for improved biomarker analysis. This presented work outlines the potential for future clinical translation of real-time Raman-based molecular imaging, by allowing easy access to patients and providing biochemical distributions from the region of interest for tissue differentiation during surgical resection.

## Materials and methods

### Raman setup

The handheld fiber-optic Raman probe, as described in our previous report^18^, is a coaxial, dual fiber handheld probe. The excitation laser is fiber-coupled into the probe through a multi-mode fiber with 105 µm core diameter, passes through a 785 nm bandpass filter to remove the silica Raman background from the excitation fiber, then through a dichroic mirror and is focused at the sample plane through an objective lens, with a working distance of 7.5 mm and a numerical aperture (NA) of 0.22, see Supp. Fig. S9. The outline of the probe can be found in the supplementary materials. The generated Raman signal is collected by the same objective lens, passes through a long-pass filter to remove the Rayleigh scattering signal, is fiber-coupled into a 200 µm fiber, which is also used as the entrance aperture of the spectrometer (Acton LS 785; Princeton Instruments, Trenton, New Jersey). The spectrometer is equipped with a back-illuminated deep depletion charged coupled device (CCD, PIXIS-400-BReXcelon; Princeton Instruments Trenton, New Jersey). A 785 nm laser with maximum output power about of 300 mW (FERGIE-785 nm laser; Princeton Instruments, Trenton, New Jersey) is used for the excitation. Taking the parameters of the diameter of the fiber, the wavelength of the laser, the grating of 830 g/mm and the type of the spectrometer into account for grating dispersion calculation, the setup has a spectral resolution of ~2.2 nm, i.e., 24 cm^−1^. The typical output laser power and acquisition time in the report was ~100 mW and 0.1 s, respectively.

### Computer vision-based positional tracking of the laser beam spot

During the acquisition, the Rayleigh scattering of excitation laser is used for positional tracking. The positional tracking method of this study has been described in detail in our previous work of ref. ^18^. In this paper, a CMOS (complementary metal-oxide-semiconductor) camera (DCC1645C; Thorlabs, Austin, Texas) replaces the conventional webcam as it has a 650 nm short-pass filter to decrease the influence of high-intensity Rayleigh scattering, which can artificially increase the detection area, decreasing the accuracy of the positional tracking. The resolution of the camera was set to 640 × 512 pixels. Briefly, in the image processing algorithm, a single plane is extracted from the color brightfield RGB image, followed by intensity thresholding. The contour of the spot is extracted by a Laplacian filter, and an ellipse contour fitting function is used to determine the center and size of the laser spot. The fitted parameters of center and size are used for Raman image reconstruction.

### Photometric stereo

Photometric stereo is used for 3D surface reconstruction and is based on estimating the surface normal by multiple conventional two-dimensional (2D) images under different illumination directions. Here, a four source photometric stereo approach^36–38^ was applied, allowing the acquisition of four 2D images under four different illumination orientations from the top, right, bottom, and left, respectively. The camera for the acquisition is identical to the one for positional tracking. Four light-emitting diodes, which are set surrounding the camera with distances of 15 cm were controlled by a multifunction I/O device (USB6001; National Instruments, Austin, Texas). The intensity value of the same pixel from these four 2D images, *I*_n_, are determined by the surface normal ***N***, the albedo (reflectivity) *k* and coordinates of four different illuminations, *L*_n_, following the equation^39^:

4 $$I_{{{\boldsymbol{n}}}} = k_{\mathrm{d}}\left( {{{{\boldsymbol{L}}}}_{{{\boldsymbol{n}}}} \cdot {{{\boldsymbol{N}}}}} \right)$$

Solving for *k*_d_***N***, i.e., $k_{\mathrm{d}}{{{\boldsymbol{N}}}} = \left( {{{{\boldsymbol{L}}}}_{\mathrm{n}}^{\mathrm{T}}{{{\boldsymbol{L}}}}_{\mathrm{n}}} \right)^{ - 1}{{{\boldsymbol{L}}}}_{\mathrm{n}}^{\mathrm{T}}I_{\mathrm{n}}$, where ***N*** is a unit vector, and the albedo *k*_d_ can be estimated as the length of this vector. With the known coordinates of the four illuminations and acquired four 2D images, the surface normal ***N*** of each pixel can be calculated, and the gradient of each pixel can be approximated as follows:

5 $$G_{{\mathrm{x,y}}} = \left(\frac{{N_{\mathrm{x}}}}{{N_{\mathrm{z}}}},\frac{{N_{\mathrm{y}}}}{{N_{\mathrm{z}}}}\right)$$

Last, the surface height map, i.e., the shape, can be iteratively approximated from a manually set start point, e.g., the center position, to the outermost pixels by integration based on the gradients between adjacent pixels.

### Fixed porcine brain sample

The porcine brain was ordered from a normal butcher shop and was isolated and fixed in 4% PFA in PBS solution overnight at 4 °C. The fixed porcine brain sample was used for the experiment after rinsing with distilled water.

### Ex-vivo tumor sample

The ex-vivo tumor samples were the center part of the biopsy resection during tumor removal surgery of muscle-skeletal sarcoma. The surrounding part which may contain conjunction part of health and tumor tissue was kept for histologic analysis. The experiment protocol was approved by Jena University, Ethik Votum 2018–115.s8.

## Supplementary information

Supplementary Information

Supplementary Video 1a

Supplementary Video 1b

Supplementary Video 2

Supplementary Video 3

Supplementary Video S1

## Data Availability

The datasets acquired for this study are available from the corresponding authors upon reasonable request.
