# Supplementary material for: Real-time molecular imaging of near-surface tissue using Raman spectroscopy
Source: Light Sci Appl. 2022 Apr 8;11:90. doi: 10.1038/s41377-022-00773-0 (PMC8993924; doi:10.1038/s41377-022-00773-0)
Supplement: Supplementary file 1 — Supplementary Information [file 41377_2022_773_MOESM1_ESM.docx]

*Supplementary information for*

**Real-time molecular imaging of near-surface tissue using Raman spectroscopy**

Wei Yang^1^, Florian Knorr^1^, Ines Latka^1^, Matthias Vogt^2^, Gunther O. Hofmann^2^, Jürgen Popp^1, 3^, and Iwan W. Schie ^1, 4, *^

^1^Leibniz Institute of Photonic Technology Jena, Albert-Einstein-Straße 9, 07745 Jena, Germany

^2^Department of Trauma, Hand and Reconstructive Surgery, University Hospital Jena, Am Klinikum 1, 07747 Jena, Germany

^3^Institute of Physical Chemistry and Abbe Center of Photonics, Friedrich-Schiller University Jena, Helmholtzweg 4, 07743 Jena, Germany

^4^Department of Medical Engineering and Biotechnology, University of Applied Sciences - Jena, Carl-Zeiss-Promenade 2, 07745 Jena, Germany

*Correspondence: Iwan W. Schie, Email: [iwan.schie@leibniz-ipht.de](mailto:iwan.schie@leibniz-ipht.de)

| **Supplementary Table S1**. Representative Raman fiber optic probe designs and reported excitation and acquisition parameters, i.e. λ: wavelength, *P*: laser power, *t*: acquisition time, *FOV*: field of view, *SR*: spatial resolution. | | | | |
| --- | --- | --- | --- | --- |
| **Probe** | | **Features** | **Typical parameters** | **Ref.** |
| **Endoscopic Raman probe** | **Volume probe** | Without any focusing optics, simplest implementation | *λ:* 785 nm, *P*: 150 mW, *t*: 1 s | [1] |
|  |  |  | *λ:* 720 nm, *P*: 7 mW, *t*: 20 s | [2] |
|  | **Confocal probe** | Addition of extra optical components, e.g. |  |  |
|  |  | - Ball lens | *λ:* 785 nm, *P*: 15 mW, *t*: 1 s | [3,4] |
|  |  | - Gradient index lens (GRIN lens) | *λ:* 830 nm, *P*: 60 mW, *t*: 1 s | [5] |
|  |  | - Aspheric lenses | *λ:* 830 nm, *P*: 34 mW, *t*: 10 s | [6] |
| **Handheld Raman probe** | | Robust, bulk, commercially available | *λ:* 785 nm, *P*: 350 mW, *t*: 0.2 s | [7] |
|  |  |  | *λ:* 785 nm, *P*: 100 mW, *t*: 2 s | [8] |
| **Fiber array-based approach** | | Imaging ability for small area |  |  |
|  |  | Fiber bundle + galvanometric scanner | *λ:* 532 nm, *FOV*: 0.07 mm^2^,  *SR*: a few µm | [9] |
|  |  | Fiber bundle + galvanometric scanner | *λ:* 785 nm, *P*: 950 mW, *t*: 0.9 s, *FOV*: 14 mm^2^, *SR*: <115 µm | [10] |

**Supplementary Figures**

**
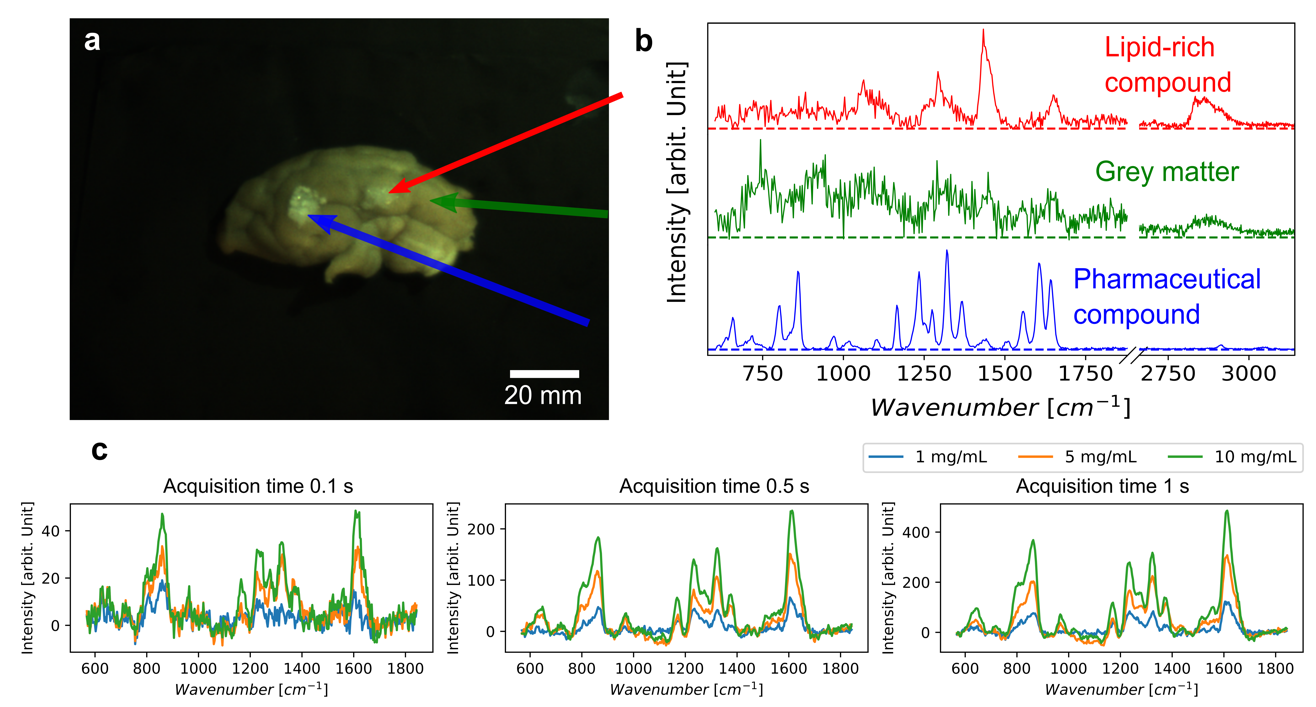
**

Fig. S1. a. Brightfield image of the 3D structured sample, i.e. a porcine brain sample where one area with a lipid-rich and one with a pharmaceutical compound are indicated. b. Relevant representative Raman spectra of the lipid-rich compound, grey matter, and pharmaceutical compound. c. Raman spectra of paracetamol solutions at different concentrations (mg mL^-1^). The solution was pipetted onto a calcium fluoride (CaF_2_) microscope slide and the probe was placed on a Z-direction manually adjustable stage with the laser focusing on the droplet. The laser power was set to approx. 100 mW, and three different acquisition time of 0.1, 0.5, 1 s were tested. The water concentration in each acquired Raman spectrum was removed through non-negative-least-squares fitting of a spectrum of the pure water^11^.


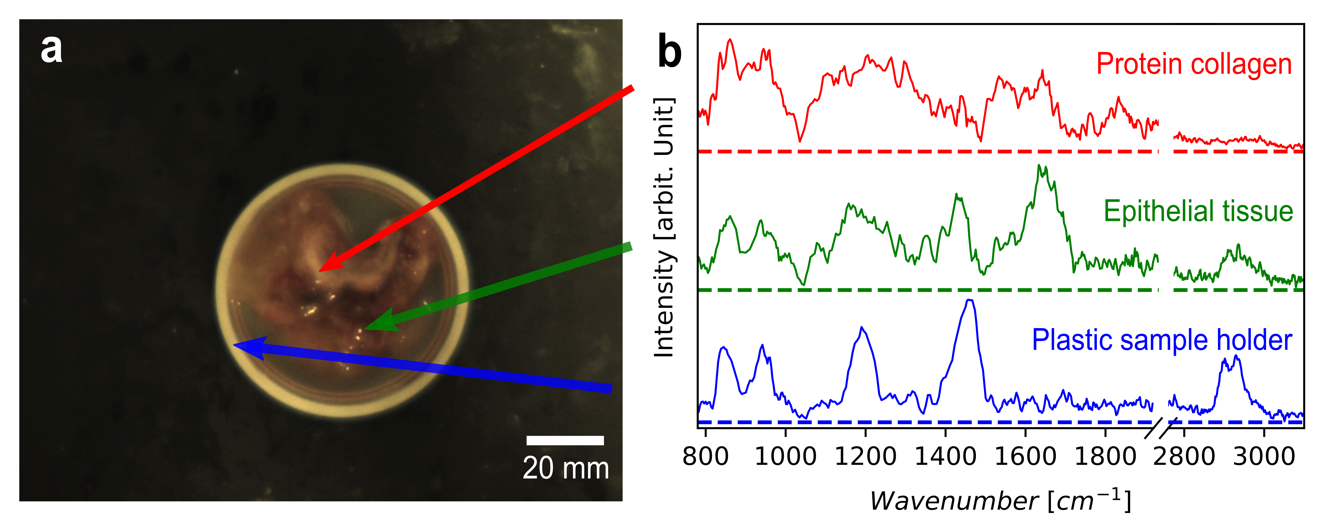


Fig. S2. a Brightfield image of an ex-vivo sample. b Representative Raman spectra of collage (red), epithel tissue (green), and plastic sample holder (blue).

Experimental results performed on an ex-vivo sample, which was resected from surgery for extensive intramuscular lipoma in the area of the pectoralis major muscle on the right for a 68-year-old patient with a size of 4 cm × 2.8 cm. The Raman image was reconstructed in about 160 s with 672 points. The brightfield image, Raman image, and the merged augmented image are shown in Figure S3a-c. The data gridding algorithm predicts the blank or missing points to fulfil the Raman image as shown in Figure S3d. The Raman image was back-projected on the sample surface which formed a live update mixed reality image to further obviously and straightforwardly present the biochemical information visually. Figure S3e and S3f show the mixed reality image of the Raman image on the ex-vivo sample with and without the data gridding and were captured by the external camera. Figure S3g shows the related Raman spectra of the chemical components. Since the sample was resected from the intramuscular lipoma, only lipid spectrum from the sample has been detected. The screen-record construction of the augmented image and video-record mixed reality by the external camera are edited to the same timeline in one video and attached as Supplementary Video S1.


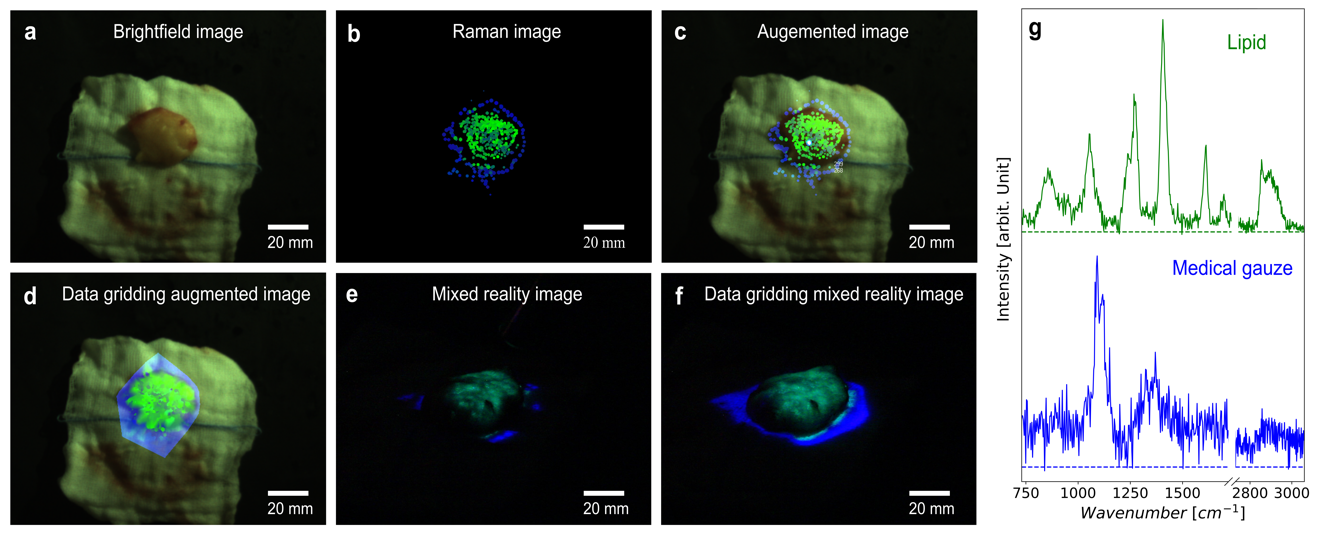


Figure S3. Representative results of the proposed approach on the ex-vivo clinical lipoma sample. a Brightfield image of the lipoma sample. b Reconstructed Raman image, with green representing the lipoma and blue the sample carrier. c Augmented molecular reality image of the lipoma sample. d Data gridding applied to the Raman image and overlaid with the brightfield image. e Back-projected Raman image on the sample, forming the mixed Raman reality image. f Interpolated mixed reality image. g The Raman spectra of different chemical components are assigned with pseudocolors, i.e. green for lipid, blue for cellulose fiber of the medical gauze.


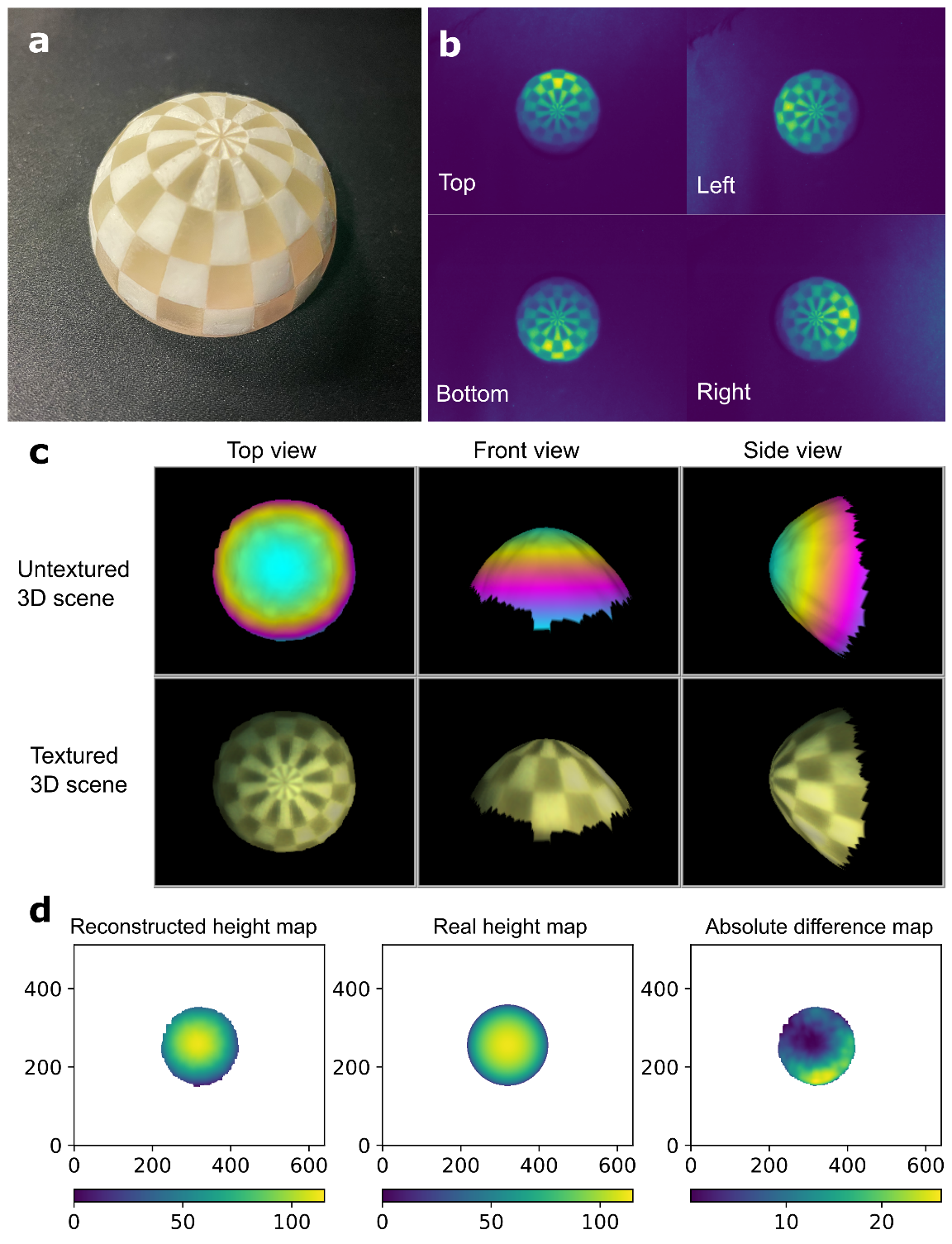


Fig. S4. 3D surface reconstruction of the hemisphere phantom. a A brightfield image of the phantom. b Intensity images under various orientations of light illumination: from the top, left, bottom, and right, respectively. c Reconstructed images under untextured 3D scene and textured 3D scene with different views: from the top, front, and side, respectively. d Height maps of the reconstructed and real hemisphere and the absolute difference between the reconstructed and real data. The real height map is plotted according to the equation: $\boldsymbol{z}\mathbf{=}\sqrt{\boldsymbol{r}^{\boldsymbol{2}}\boldsymbol{-}\left( \boldsymbol{x-320} \right)^{\boldsymbol{2}}\boldsymbol{-}\left( \boldsymbol{y-256} \right)^{\boldsymbol{2}}\boldsymbol{+b}}$, here *r* = 104, (25 mm ÷ 0.24 mm pixel^-1^) is the radius of the hemisphere, (320, 256) is the center of the image and *b* = 8.3 (2 mm) in the lower part of the phantom. The root-mean-square error (RMSE) between reconstructed and real is ~3.7 pixels (0.9 mm) and the normalized root-mean-square error (NRMSE) is 3.3%.

**
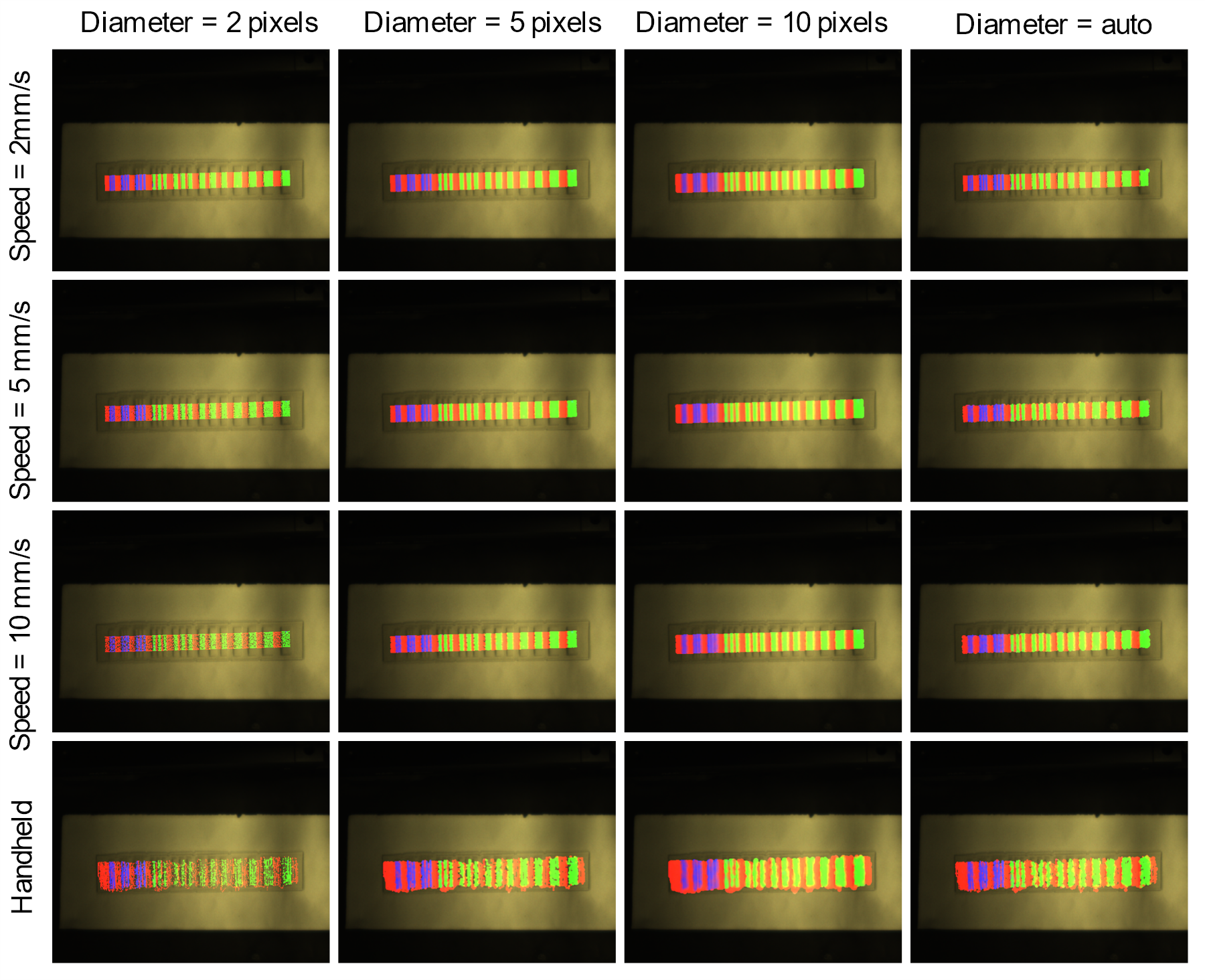
**

Fig. S5. Evaluation of the influence of scanning speed and mapping dimension on the spatial resolution and comparison to handheld operation. Raman images in combination with augmented reality were acquired automatically with a translational stage at various speeds, showing that for an increasing of the speed the fill-factor is reduced for a fixed diameter size. This can be improved by dynamically adjusting the circle diameter during the acquisition, resulting in an improved visualization of the molecular distribution during the real-time data acquisition and analysis. The reconstruction with different sizes of diameter and auto-scale according to the rules of Eq. (3).

**
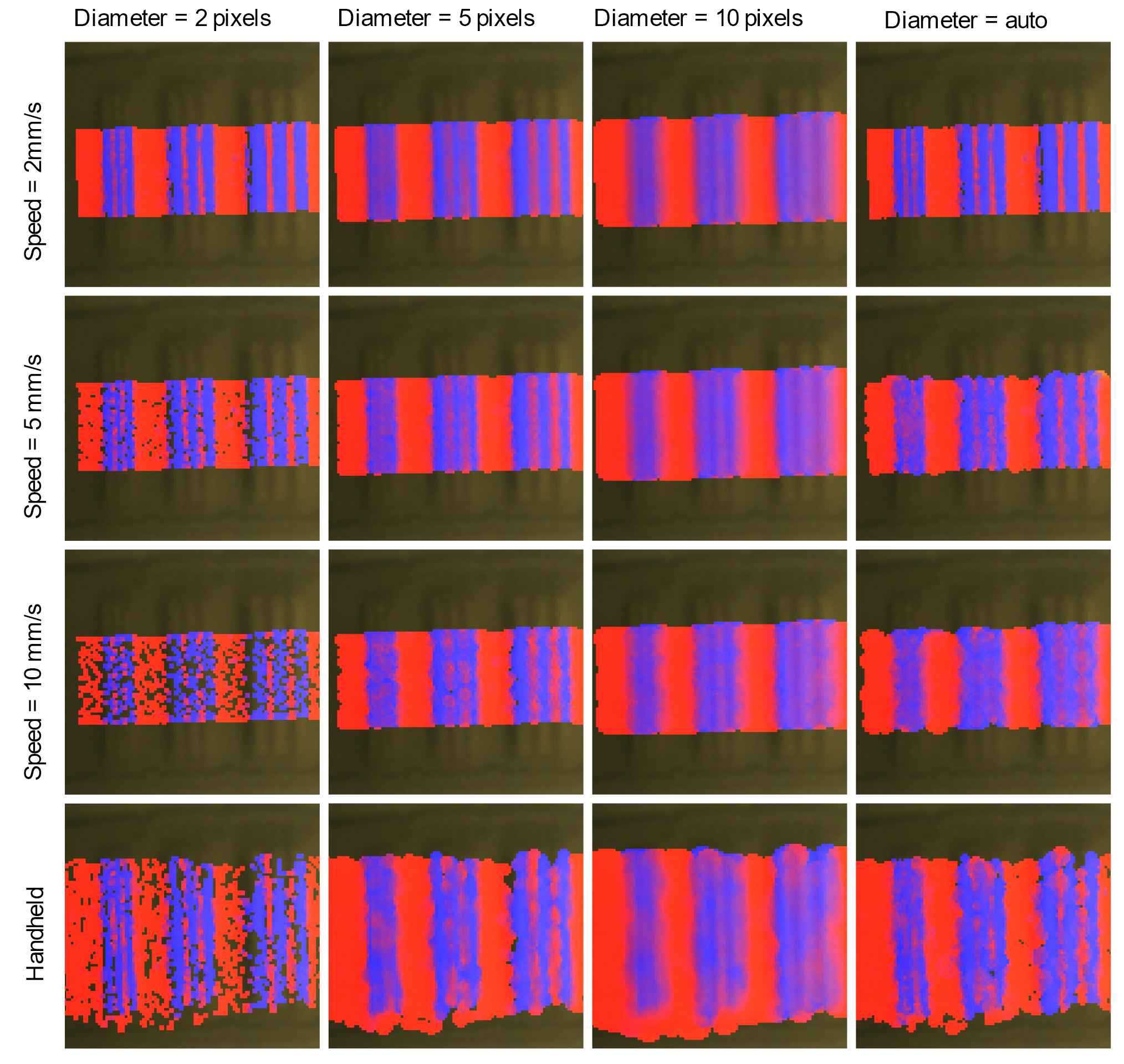
**

Fig. S6. Zoomed-in images for ROI outlined in Fig. S5, indicating the details for the resolution measurements.

**Projector-camera calibration for mixed reality display**

A coordinate checkerboard-based projector-camera calibration is applied for realizing the back-projecting of the molecular information on the sample^12–14^. The calibration flowchart is presented in Figure S7. A checkerboard image with 31 × 23 grid was created and projected on the sample plane by a projector and captured by the brightfield camera. For establishing the correlation function between the OpenCV^15^ function cv2.findChessboardCorners( ) was applied to both, the original checkerboard image and the captured image. The detected positions of the corners were identified and displayed on both images as red dots. By applying the OpenCV function cv2. findHomography( ) to the detected points, a homographic matrix can be built to connect the projector and the camera, which indicates the rotation and translation between the projected image and the captured image.

**
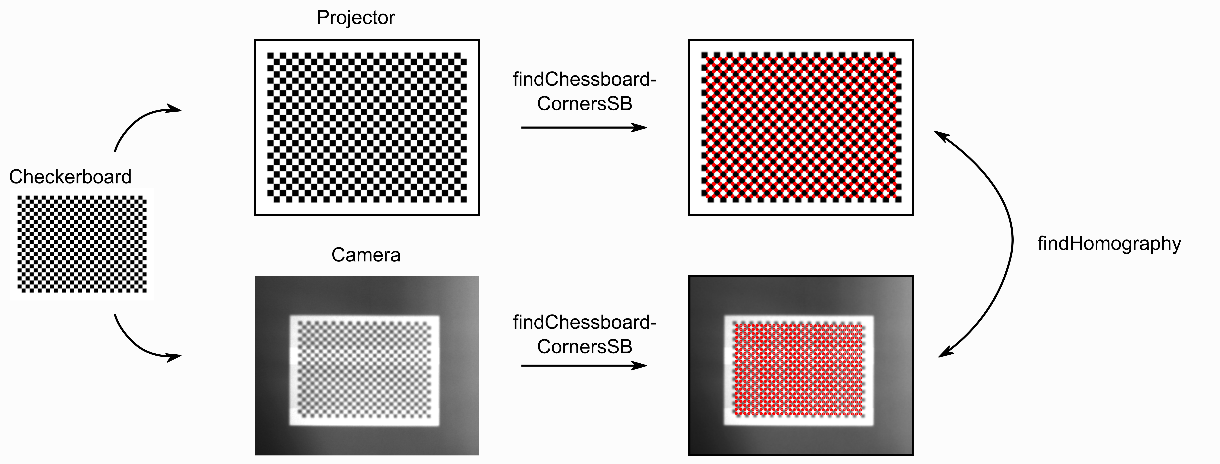
**

Fig. S7. The flowchart of projector-camera calibration.

Based on the homographic relationship, it is possible to transform the reconstructed molecular image to map it onto the sample. The performance was evaluated on five manually set points, which represent possible locations for acquire Raman data, in a test image, Figure 8Sa. Then, by applying the homographic matrix approach the coordinate locations can be transformed to match the checkerboard image, Figure S8b. By projecting the converted image onto the surface of the worktable, capturing the information by a brightfield camera, and determining the point positions it is possible to establish the deviation, Figure S8c. Comparing the points positions between S8a and S8c, one can get a root mean square error (RMSE) of ~1.5 pixels, which corresponds to normalized error of 0.3%, relative to the image size. Taking the spatial resolution of 0.24 mm per pixel (640 × 512 pixels for a field of view of ~15.6 cm × 12.5 cm) into account, there is a real mismatch of ~0.5 mm between the projected image and the real sample which is acceptable for the current implementation.


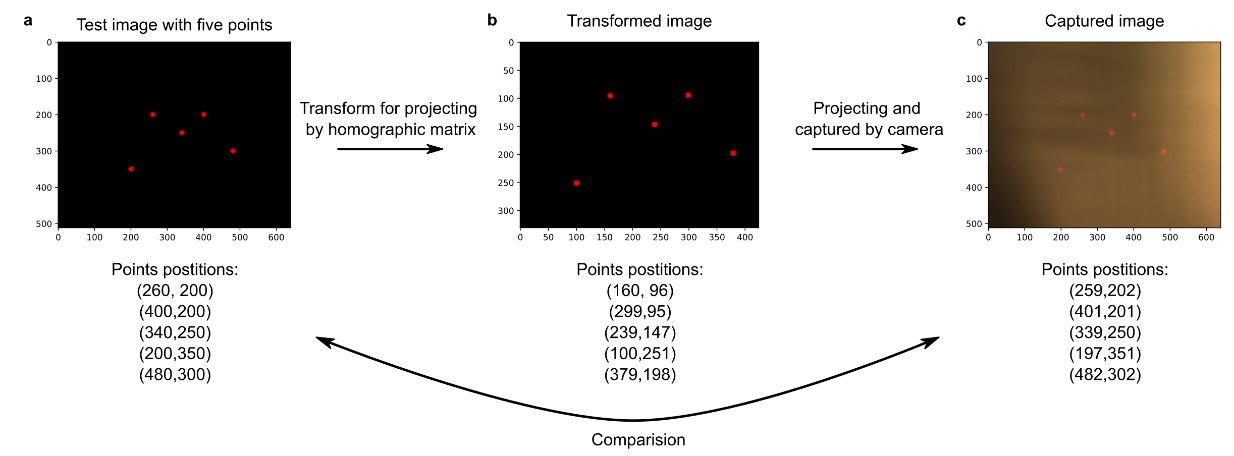


Fig. S8. Evaluation of projector-camera calibration by using five points. a Manually setting of five points as a test image and related positions. b Transformation of the original test image to the projection image by homographic matrix and related transformed points positions, and c, the real captured image by the camera and related points positions.

**Diagram of the probe**

**
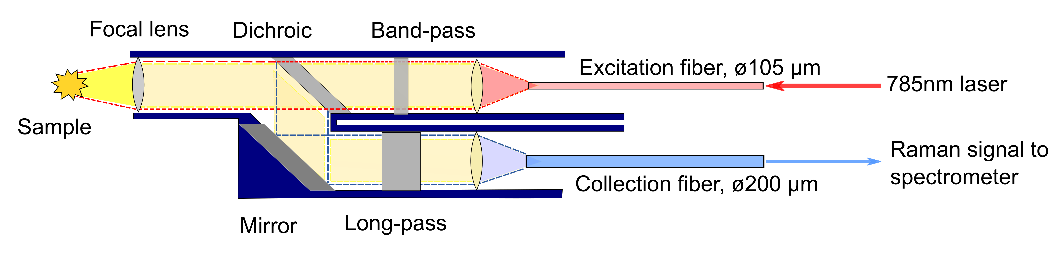
**

Fig. S9. The optical design of the handheld fiber-optic probe. The excitation laser is fiber-coupled through a 105 µm multi-mode fiber and passes through a 785 nm bandpass filter to remove the silica Raman background generated in the excitation fiber. After passing a dichroic mirror it is focus to the sample plane though an objective lens with a working distance of 7.5 mm and a numerical aperture (NA) of 0.22. The generated Raman signal is collected by the same objective lens, passes through a long-pass filter to remove the Rayleigh scattering signal and fiber-coupled into a 200 µm fiber, which is also used as the entrance aperture of the spectrometer.

**Information of the supplementary videos:**

**Supplementary Video 1a.** The 3D scene view record of the 3D structured sample in individual windows of non-textured height image, original color textured image, and augmented chemical textured image.

**Supplementary Video 1b.** The computer screen and mixed reality result of the molecular imaging for the 3D structured sample surface was screen-recorded and video-recorded by the external camera.

**Supplementary Video 2.** The computer screen of the Raman imaging for the ex-vivo sample was screen-recorded.

**Supplementary Video 3.** The 3D scene view record of the hemisphere phantom in individual panels of non-textured height image, original color textured image, Raman textured height image, and augmented chemical textured height image.

**Supplementary Video S1.** The computer screen and mixed reality result of the molecular imaging for the extra ex-vivo sample was screen-recorded and video-recorded by the external camera.

**Reference**

1 Shim MG, Wilson BC, Marple E, Wach M. Study of Fiber-Optic Probes for in vivo Medical Raman Spectroscopy. *Appl Spectrosc* 1999; **53**: 619–627.

2 Komachi Y, Sato H, Aizawa K, Tashiro H. Micro-optical fiber probe for use in an intravascular Raman endoscope. *Appl Opt* 2005; **44**: 4722.

3 Motz JT, Hunter M, Galindo LH, Gardecki JA, Kramer JR, Dasari RR *et al.* Optical Fiber Probe for Biomedical Raman Spectroscopy. *Appl Opt* 2004; **43**: 542.

4 Mo J, Zheng W, Huang Z. Fiber-optic Raman probe couples ball lens for depth-selected Raman measurements of epithelial tissue. *Biomed Opt Express* 2010; **1**: 17.

5 Almond LM, Hutchings J, Kendall C, Day JCC, Stevens OAC, Lloyd GR *et al.* Assessment of a custom-built Raman spectroscopic probe for diagnosis of early oesophageal neoplasia. *J Biomed Opt* 2012; **17**: 0814211.

6 Day JCC, Bennett R, Smith B, Kendall C, Hutchings J, Meaden GM *et al.* A miniature confocal Raman probe for endoscopic use. *Phys Med Biol* 2009; **54**: 7077–7087.

7 Zhang Y, Liu H, Tang J, Li Z, Zhou X, Zhang R *et al.* Noninvasively Imaging Subcutaneous Tumor Xenograft by a Handheld Raman Detector, with the Assistance of an Optical Clearing Agent. *ACS Appl Mater Interfaces* 2017; **9**: 17769–17776.

8 Karabeber H, Huang R, Iacono P, Samii JM, Pitter K, Holland EC *et al.* Guiding Brain Tumor Resection Using Surface-Enhanced Raman Scattering Nanoparticles and a Hand-Held Raman Scanner. *ACS Nano* 2014; **8**: 9755–9766.

9 Doronina-Amitonova L V., Fedotov I V., Fedotov AB, Zheltikov AM. High-resolution wide-field Raman imaging through a fiber bundle. *Appl Phys Lett* 2013; **102**: 161113.

10 St-Arnaud K, Aubertin K, Strupler M, Madore WJ, Grosset AA, Petrecca K *et al.* Development and characterization of a handheld hyperspectral Raman imaging probe system for molecular characterization of tissue on mesoscopic scales. *Med Phys* 2018; **45**: 328–339.

11 Duraipandian S, Knopp MM, Pollard MR, Kerdoncuff H, Petersen JC, Müllertz A. A fast and novel internal calibration method for quantitative Raman measurements on aqueous solutions. *Anal Methods* 2018; **10**: 3589–3593.

12 Zhang Z. A flexible new technique for camera calibration. *IEEE Trans Pattern Anal Mach Intell* 2000; **22**: 1330–1334.

13 Chum O, Pajdla T, Sturm P. The geometric error for homographies. *Comput Vis Image Underst* 2005; **97**: 86–102.

14 Moreno D, Taubin G. Simple, accurate, and robust projector-camera calibration. *Proc - 2nd Jt 3DIM/3DPVT Conf 3D Imaging, Model Process Vis Transm 3DIMPVT 2012* 2012; : 464–471.

15 OpenCV Tutorials. https://docs.opencv.org/master/d9/dab/tutorial_homography.html.
